# Mastering Quality: Uniting Risk Assessment With Quality by Design (QbD) Principles for Pharmaceutical Excellence

**DOI:** 10.7759/cureus.68215

**Published:** 2024-08-30

**Authors:** Abdul M Kaleem, Jebastin Koilpillai, Damodharan Narayanasamy

**Affiliations:** 1 Pharmacy, SRM Institute of Science and Technology, Chennai, IND; 2 Pharmaceutics, SRM Institute of Science and Technology, Chennai, IND

**Keywords:** risk assessment tools, quality by design, quality control, pharmaceutical development, risk management

## Abstract

The aim of this article is to present ideas and tools of risk assessment that can be implemented to overcome various pharmaceutical quality challenges. These elements cover the development, production, distribution, inspection, and reporting of review procedures for drug substances, drug products, and biological products at every stage of their lifecycle.

In light of the constant requirement to ensure the drug's efficacy, safety, and quality, the pharmaceutical sector is always evolving. A key strategy for attaining pharmaceutical excellence in this dynamic environment is incorporating novel methods like quality by design (QbD) and risk assessment. Risk assessment is a methodical strategy to locate, assess, and minimize any risks in the manufacturing and development of pharmaceuticals. On the other hand, QbD principles place more emphasis on end-product testing and place it in the context of designing quality into the product from the beginning.

The major goal of this paper is to address and examine the integration of risk assessment approaches with the QbD principle workflow to ensure pharmaceutical quality. Recent articles on how risk assessment has been used in pharmaceuticals were evaluated. To provide a useful overview, the various assessment methodologies have been highlighted, emphasizing their benefits and drawbacks.

## Introduction and background

A global regulatory strategy known as "quality by design" (QbD) tries to enhance pharmaceutical development by establishing manufacturing processes and controls in advance to ensure that the required product performance is consistently produced. The International Conference on Harmonization (ICH) guidance provides a description of the pharmaceutical development principles that are pertinent to QbD (ICHQ8-11). Improved process and product understanding can only be achieved through science- and risk-based activities that make use of appropriate techniques and resources. Although the pharmaceutical sector is quickly implementing QbD concepts to create safe, effective, and high-quality medications, we are still in the process of accumulating all the data and experience needed to link and currently still working on showing the advantages of QbD to all parties involved [1]. In the near future, the optimization of sophisticated drug delivery systems will benefit from an understanding of the formulation and process parameters via the lens of QbD. The process includes several phases, including determining the product features that have a significant impact on the product's safety and/or efficacy, as indicated by the quality targeted product profile (QTPP) and critical quality attributes (CQA). Its goal is to manage the desired product quality profiles within the framework of a certain set of quality metric. Risk assessment concepts, when applied effectively, are recognized to reduce the impact of hazards on any given project. However, it is typically unclear whether crucial success determinants provide a baseline threat to pharmaceutical project success. There are three steps in the risk assessment procedure. To identify the risks and gain a better understanding of them, a list of potential risks associated with the target process is generated in the first phase: either concurrently, qualitatively, or numerically. Lastly, comes the decision-making stage, wherein it is decided whether to take on more or less risk. After risk assessment, risks will be evaluated to see whether the action performed had a positive or negative impact on the final result. According to ICHQ9, risks should be shared with all parties involved at every stage of the risk management procedure. In the pharmaceutical industry, risk assessment is vital to ensuring the safety, efficacy, and quality of the medicinal products. Risk assessment in the pharmaceutical industry is done to safeguard public health by detecting and mitigating hazards that may affect the effectiveness, safety, and quality of medications [2]. Ensuring that products satisfy high quality standards, minimizing any negative impacts, and achieving regulatory compliance all depend on this procedure. Over the past few decades, risk assessment approaches in the pharmaceutical business have made tremendous progress. In the past, the sector depended on reactive strategies, taking action against hazards only after they materialized. But the demand for a more methodical and proactive approach has prompted the creation and acceptance of sophisticated risk management techniques. Technological developments, better knowledge of intricate biological systems, and higher regulatory standards are the driving forces behind these strategies. A wide range of rigorous and thorough qualitative and quantitative methodologies are used in modern risk assessment procedures.

## Review

Risk assessment techniques in pharmaceutical development

FMEA

"Drug-related problems" refer to issues involving medications and include both intrinsic and extrinsic toxicities. Also referred to as adverse drug reaction, intrinsic toxicity is the result of a medication's pharmacological, chemical, and/or pharmaceutical properties interacting with the human biosystem. Improving patient safety requires identifying these pharmaceutical errors and figuring out how to keep them from happening again. The most important way to guarantee patient safety in healthcare settings is to promote a safety culture. The third worldwide Patient Safety Challenge (2017) aims to address systemic deficiencies in healthcare systems in order to prevent pharmaceutical errors on a worldwide scale. The right processes and systems must be in place to enable the creation of an effective safety culture. Failure modes and effects analysis (FMEA) is an important risk assessment technique in pharmaceutical manufacturing. This approach detects probable failure modes in operations, goods, or systems, assesses their impact on the system, and prioritizes risk mitigation actions. The process begins with establishing the FMEA's objectives and scope, subsequently forming a cross-functional team and mapping out the full manufacturing process. Each phase of the process is examined to discover potential failure mechanisms using brainstorming and historical data. The consequences of such failures are evaluated for their influence on the quality of the product, safety for patients, and compliance with regulations, with their severity, frequency, and identification ranked on a predetermined scale. This Risk Priority Number (RPN) is used to rank failure scenarios according to their risk levels. Purpose quality management technologies like FMEA are employed in a variety of industries to enhance quality. This research attempts to show that FMEA may be used as a performance enhancement technique based on a case study of process improvement for a drug development project.

*Design/methodology/approach*: The proposed FMEA process has several key features, including an interface that makes it simple to visualize complex processes in pharmaceutical research, the detection of undesirable effects as evidence of process defects, and a numerical estimation of the negative impacts associated with quality and efficiency.

**Figure 1:**
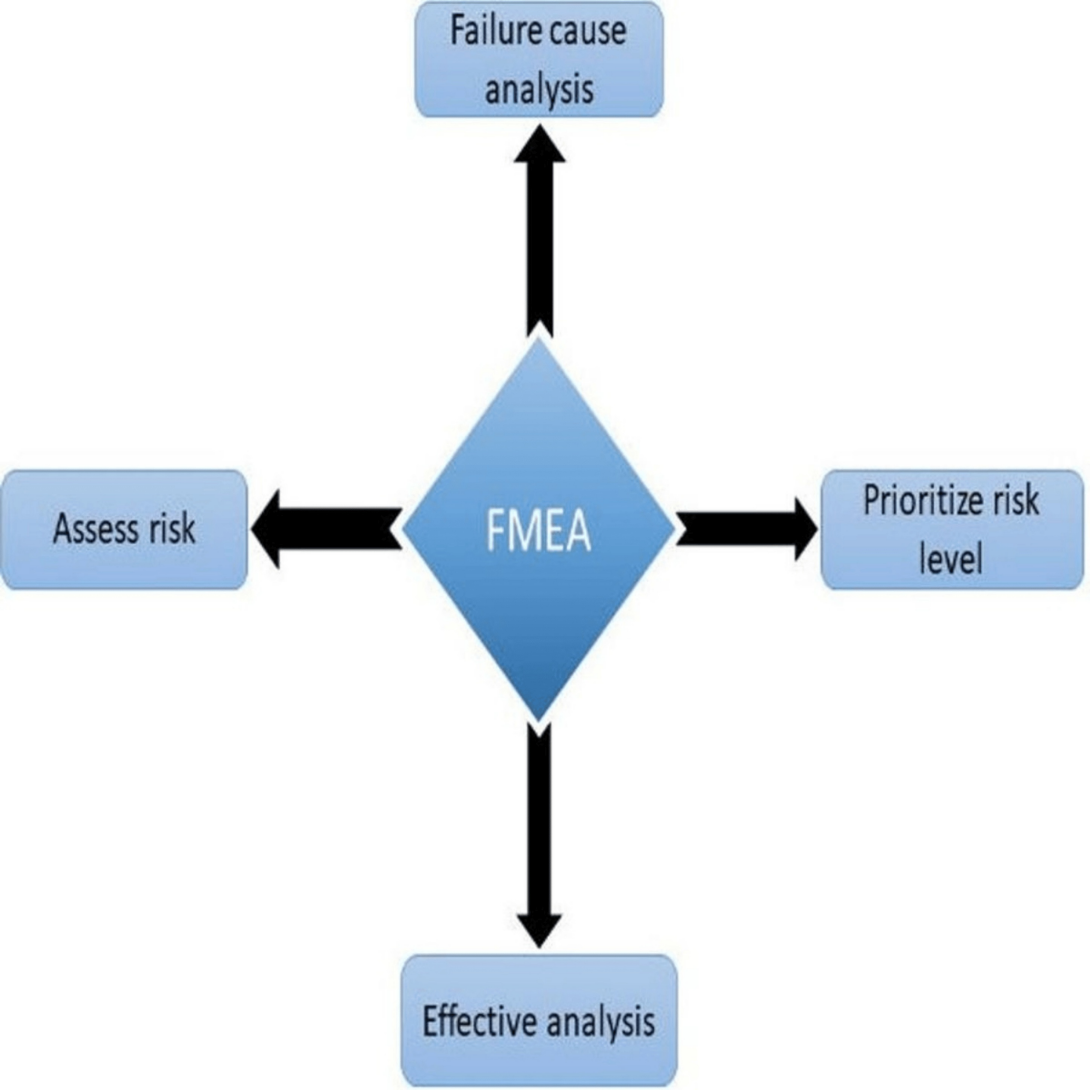
Various elements of FMEA This image was created by Dr. Abdul M. Kaleem. FMEA: Failure modes and effects analysis

*Findings:* The efficiency of the proposed FMEA approach was assessed using in vivo screening/profiling throughout early drug discovery. A flow diagram was used to visually represent the process that needed to be improved [3]. This evaluation will cover topics such as the makeup of the groups involved in FMEA procedures, the benefits and outcomes of FMEA in healthcare settings, and the techniques for analyzing FMEA results. In order to enhance drug safety, the current study also examines participants' and researchers' perspectives regarding the use of FMEA for a specific healthcare practice. Various elements of FMEA are mentioned in Figure 1.

FMECA

The pharmaceutical sector frequently utilizes failure modes, effects, and criticality analysis (FMECA), a systematic and structured technique to improve process reliability, product quality, and patient safety. As part of FMECA, processes are evaluated to identify the possible failure modes, their causes, and effects. The criticality of these failures in the manufacturing process or product quality is also determined. The main goal is to proactively find and fix the possible problems before they influence the finished product or patient safety. Failure modes are rated according to severity, occurrence, and detection in FMECA [4]. The severity rating, which is usually on a scale from 1 to 10, with 10 denoting the most severe impact, evaluates the possible impact of a failure mode on patient safety, product quality, and regulatory compliance. The occurrence rating, which likewise runs from 1 to 10, ranks the probability of a failure mode occurring, with 10 denoting extremely likely occurrences. With a score of 10 representing the least successful detection techniques, the detection rating assesses how well the current controls are able to identify failures before they influence the process or product. Process deviations can be decreased by 25% and equipment failures by 30% by utilizing FMECA. Because there have been fewer recalls and reworks, companies have reported cost savings of up to 20%. Improved regulatory compliance has also been noted, as evidenced by a 15% drop in audit findings pertaining to manufacturing procedures. These figures highlight the importance of FMECA in upholding operational effectiveness and high levels of quality [2]. The primary metrics that need to be calculated are as follows:

Occurrence (O): Which represents the likelihood that a failure mode will occur?

Severity (S): Which represents the worst-case scenario of a failure based on the degree of harm, property damage, or system damage that could ultimately occur?

Detection (D): The ability to look into possible reasons why a failure mode might occur.

FMECA is closely related to FMEA. Every tool seeks to identify the failure modes that may result in a failing product or process. While FMECA uses quantitative data from an entity with established failure frequencies, FMEA is subjective and investigates "what-if scenarios."

HACCP

Hazard analysis and critical control points (HACCP), a proactive strategy that identifies and evaluates potential hazards in the production of pharmaceuticals or healthcare products, establishes control measures to prevent or eliminate these hazards, ensuring reliability and also safety of the final product. This systematic approach involves detecting, assessing, and monitoring safety hazards throughout the production process to minimize risks associated with the identified hazards during pharmaceutical manufacturing. Quality hazards are controlled by the validating critical operations and processes through Good Manufacturing Practices (GMP), ensuring compliance and safeguarding manufacturing staff [5]. The key objective of the HACCP is to produce safety products while minimizing or eliminating the need for time-consuming endpoint testing. Before the implementation of HACCP, many companies relied on endpoint testing to determine product acceptability. HACCP aims to reduce endpoint tests by conducting inspections during the process and assessing risks at every phase to determine appropriate actions in case of significant hazards [1]. HACCP is an organized, proactive, and preventive approach to ensuring product quality, dependability, and safety. HACCP includes the subsequent seven steps: (i) perform a hazard analysis to determine preventive actions for each stage of the process, (ii) identify the important control points, (iii) establish essential limits, (iv) set up a mechanism to monitor the essential control points, (v) establish corrective measures for when monitoring shows that essential controls are not under control, (vi) check that the HACCP procedure is working properly, and (vii) establish a record-keeping system.

HACCP principles

Analysis of Hazard

Hazard analysis is defined as an approach in which information on hazards is acquired and assessed, as well as circumstances which lead to the development of these hazards, with the goal of determining whether hazards are serious enough to be included in the HACCP plan. During hazard analysis, three types of dangers must be examined: biological, chemical, and physical. It is advised to do a two-stage risk assessment.

Establishing Crucial Control Points

Determining CCPs is the very next HACCP principle after the hazard analysis. CCP is the point at which control could be applied as thought to be essential for preventing, eliminating, and bringing a risk within acceptable boundaries. CCP is the final step in hazard control before shipment. Furthermore, CCP denotes the moment in time at which significant danger arises from a loss of control.

Determine the Critical Limit

Once the CCPs have been identified, critical limitations need to be set for each phase that is a CCP. Critical boundaries are determined based on past data or degree of regulatory action. In some cases, more than one the critical limit at a particular stage will be required. Criteria like temperature, water activity, and time are frequently used. When defining critical limits, the HACCP team needs to exercise caution and make sure they are as realistic as feasible for the process at hand. Establishing crucial limits helps a control system and suitable monitoring to be implemented.

Construct a Monitoring Framework

The concept focuses on choosing a useful method for keeping an eye on each CCP and critical limit. Observing each unique CCP becomes extremely important since it ensures that CCPs are compliant and do not exceed the vital limits. Monitoring techniques should identify control loss at the CCP, and information of this kind should ideally be given promptly so that repairs can be made to ensure process control and prevent violations of key limits. The critical limit is observed either continuously or sporadically. It would be possible to design a computer system for measurement at regular intervals. One must keep an eye on the system or even calibrate it regularly to ensure that it operates effectively.

Implement Corrective Actions

Since there is no such thing as an ideal approach, if preventive measures don't work, remedial action should be taken. with the intention of appropriate remedial actions ought to be devised for every CCP to handle deviations whenever they occur.

Qualitative risk assessment

The systematic, science-based method of estimating a risk's likelihood, severity, and accompanying consequences is known as qualitative risk assessment [6]. A risk manager might benefit from qualitative hazard identification or risk assessment when determining priorities and making decisions on policies, including how much money to spend on sampling. Certain qualitative evaluations can be used as instruments to determine and rank the needs for further research as well as to add to the body of published work on risk analysis. Qualitative risk analysis is an approach of evaluating and arranging found risks based on their significance and likelihood of ramifications. The goal of the qualitative risk analysis is to generate a selection of dangers that should be prioritized over others.

Quantitative risk assessment

Many organizations recognize the four primary elements of risk assessment as hazard recognition, assessment of exposure, dose-response assessment (or hazard characterization), and risk characterization [6]. Risk analysis connects risk management and risk communication to risk assessment. A comprehensive quantitative risk assessment might not be possible due to limitations in data quality, time, staff, or resources. With an emphasis on letting the data speak, careful data analysis, formal inferencing, and comprehensive documenting of variability and uncertainty, data gaps are not always a barrier to quantitative risk assessment.

Quality risk management

One of the most recent techniques for process quality management is quality risk management (QRM). This offers substantial advantages in terms of determining the important risks and establishing control over them. By focusing on products and processes, the pharmaceutical industry can ensure that the proper resources are employed at the appropriate moment in order to improve product and process quality [7]. Early in the twenty-first century, the pharmaceutical product business experienced technological advancement due to a shift in legislation that resulted in a change in the idea of product quality. A new vision for ensuring product quality consequently arose, emphasizing an integrated approach to quality risk management procedure for identifying, evaluating, communicating, and reviewing the risks to the quality of drug production. It is a way to back fact-based quality management decisions and satisfy legal requirements in a validation process, which verifies that the drug manufacturing processes meet the highest standards of quality [2].

Qualitative Risk Management Process

The process is divided into four basic phases, beginning with risk assessment and ending with a review to determine the extent to which these risks affect the production procedure and where the essential improvement efforts should be focused. The phases of the quality risk management process comprise the subsequent stages: quality risk assessment, quality risk control, quality risk communication, and quality risk review.

Quality Risk Assessment

It uses analytical techniques and historical data to perform risk evaluations. The process of risk assessment involves several steps in addition to comparing the identification of a risk and its analysis to pre-established standards.

*Risk evaluation:* A critical component of the QRM process is the identification of quality risk sources according to user feedback. This facilitates the application of statistical methods and analyses relevant to the understanding of risk sources.

*Quality risk analysis:* This step helps build a plan of action for constant enhancement of quality by assessing the production process's strengths and shortcomings and then selecting the most appropriate modifications. It also aids in statistically assessing the link between the system itself and patient safety. Quality risk evaluation is the process of measuring risk utilizing data and information from previously studied factors using analytical techniques.

At the moment, risks can be described quantitatively using numerical probability or qualitatively using data on the factors that have been analyzed using analytical techniques. Risks can be statistically expressed using a numerical probability or qualitatively described using a risk expression.

Quality Risk Control

Determining whether to take on or minimize risks is known as quality control. In order to reduce risks, this step aims to determine whether risks should be accepted or refused at this point and what steps can be taken to accomplish so.

*Risk reduction:* Actions required to decrease the possibility of harm and its occurrence are known as risk reduction. Activity conformance to quality requirements is commanded in this step.

*Risk acceptance:* Accepting the danger entails making this decision. The judgment will be right if the risk is within acceptable bounds, even though the dangers might still exist despite the use of effective risk management techniques. When accepting a risk that exceeds reasonable bounds, one may have made the incorrect choice in this instance.

Quality Risk Communication

Effective risk communication involves informing stakeholders about particular and possible hazards as well as how they have been managed. This can be done in an official or informal manner.

Quality Risk Review

It is a frequent and continuous review of QRM practices. This step focuses on reviewing the earlier phases with a focus on quality. It also entails ongoing review and evaluation (feedback) to ascertain the degree to which the intended goals (outputs) are met and to make any necessary changes to any or all system components in response to information from stakeholders or customers.

Event tree analysis (ETA)

In order to determine the possibility and frequency of loss and derivative incidents, the quantitative risk assessment (QRA) model uses probability calculations. In order to determine the number and composition of those participating, it also computes the influence range. In quantitative analysis, loss evaluations include basic financial losses due to personnel, explicit financial loss due to material, and overall economic loss [8]. Plotting graphs to compare the standards with predicted risks allows for an examination of acceptable risk levels. In light of the stated reasons, poisoning via toxic releases is therefore more dangerous than fire and explosion. Following the discovery of this risk, a reasonable proposal was made to withdraw or decrease it in order to keep it within a reasonable range. An important first step in consequence analysis is predicting release rates. PHAST software (DNV, Bærum, Norway) was used to determine the acid gas dispersion modeling by varying a number of parameters. Probit functions can be used to quantify effects.

Key Points of Event Tree Analysis

What is the chance and potential course of events in the case that ClO_2_ is released following the chemical selection process utilizing Toxicity Index numbering? Event tree analysis (ETA) is one method that can be used in this analysis. Typically, branches are drawn to the right and the starting event is positioned on the left. There should only be a safety function in the branches [7]. Intermediate events can be separated into more than two categories as long as they are mutually exclusive, which means they cannot occur simultaneously. Intermediate events are typically split into a binary (success/failure or yes/no). If a spark is the initial event, there is a chance that the spark will ignite a fire, which may be expressed as a binary yes or no, and there is also a chance that the fire will spread to a tree or not. End states are categorized into groups based on their severity of repercussions or level of success.

Methodology

The primary goal of event tree analysis is to determine the probability of potential undesirable effects that may develop as a result of the specified beginning event and cause harm. To construct the event tree diagram, a thorough understanding of the system is required to understand the beginning events, accident chances, and intermediate events [1]. The initiating event initiates the event tree, and all the following events are mixed (successful or unsuccessful). Every event generates a trajectory that can be utilized for assessing the overall probability of a series of either success or failure along that path. Fault tree analysis may be utilized to predict intermediate event failures as well as success probabilities.

Steps to Perform an Event Tree Analysis

Describe the structure of the system: Specify what has to be done and where the limits should be drawn.

Determining the accident scenarios: To identify hazards or possible mistakes in the system layout, do a system evaluation.

Determining the initiation events: To determine the initiating events, do a hazard analysis.

Determine an intermediate event: Determine the measures related to a particular situation.

Create the event tree diagram.

Determine event failure chances: Fault tree analysis can also be used to determine the failure probability if it cannot be acquired.

Determine the risk associated: With the outcome by measuring the overall probability of the possible event routes.

Analyze the risk associated with the result: Analyze each route risk and decide whether it is acceptable or not.

Suggestion for corrective action: If the predicted risk of a path is unacceptable, make design changes to reduce the risk.

Record the ETA: Use event tree diagrams to capture the entire workflow and make any necessary adjustments for new data.

Bowtie analysis

The bowtie technique was adopted by the Royal Dutch Shell Group as the company standard for risk analysis and management in the early 1990s. Based on their best practices, Shell carried out a thorough investigation of the bowtie method's use and created stringent guidelines for the description of every section. The need to make sure that proper risk barriers were in place throughout all of its global activities served as Shell's main driving force. The oil and gas industry quickly adopted the bowtie technique following Shell since the diagrams made risk management procedures easier to see and understand. The bowtie technique has become popular in the mining, aviation, chemical, maritime, and healthcare sectors over the past 10 years. Uncontrolled discharge of utilities, cleaning supplies, and intermediate/final products is another risk that can seriously harm the nearby or surrounding environment. Inadequate plant operation and maintenance can also lead to expensive equipment replacement or repair costs during downtime. Finally, in the era of social media, patient safety is a critical factor that influences both reputation and quality [9]. Translating this linear (one-dimensional) model into a two-dimensional format yields a comprehensive overview of the scenario, with barriers positioned appropriately between the top event (where/when containment or loss of control occurs) and between the consequences (where effects are realized) of the top event. Using visual aids rather than written ones to convey a risk assessment makes it easier to identify weak points, such as threats or obstacles, that need closer examination or improvement.

Bow Tie Analysis in Healthcare Applications

**Figure 2:**
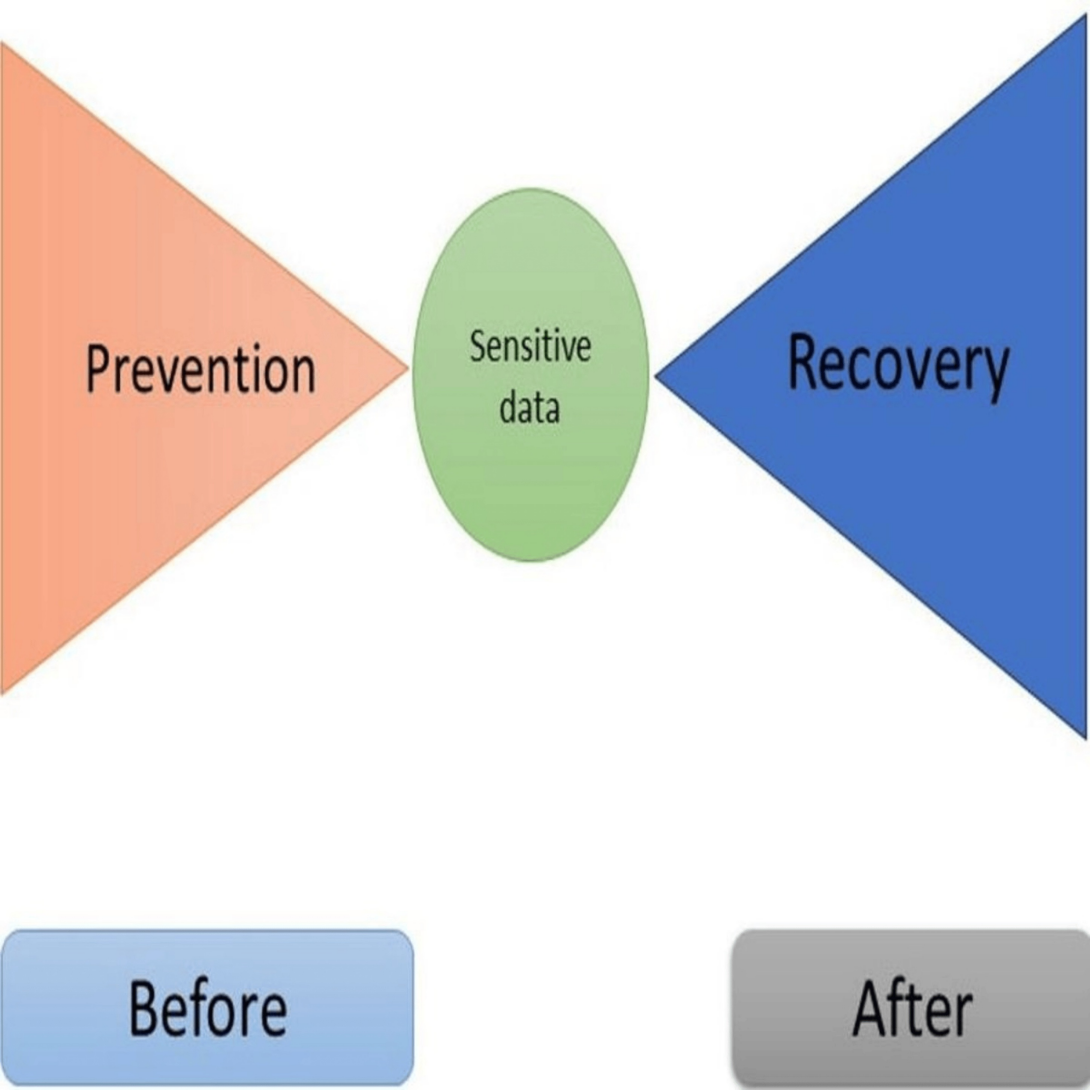
The graphical representation of bow tie analysis This image was created by Dr. Abdul M. Kaleem.

This section explains how intensive care units (ICUs) employ bowtie analysis to maintain patient safety. The study's dangers of emphasis included various conditions like medication events, infections, and pneumonia that could have a detrimental effect on patient safety. In this review, emphasis was placed on the viability and practicality of adding additional barriers. Additionally, the scientists noted that bow tie analysis was reasonably simple to do, which enhanced ICU staff acceptance. The graphical representation of bowtie analysis is mentioned in (Figure 2).

Preliminary hazard analysis (PHA)

In the initial phases of the production process, when the medical device layout is still unresolved, PHA is a technique for identifying the risks, hazardous circumstances, and incidents that can cause harm. PHAs are typically used as the foundation for a more in-depth study. The team of designers may organize and focus effort on the riskiest elements of the system by utilizing the PHA's advanced knowledge to pinpoint the safety-sensitive components of the system concept and assess the possible hazards associated with the entire system. A qualitative analysis is used to identify all the potential risks and unintentional occurrences that could lead to an accident [10]. The unforeseen occurrences are ranked in order of severity, and essential risk mitigation and subsequent actions are determined. PHA is also referred to as Hazard Identification (HAZID) or Rapid Risk Ranking. It is used during the first stages of a project (such as plant setup). During PHA analysis, it is necessary to consider hazardous elements, safety, environmental limits, functionality of the system, infrastructure, machinery, people proficiency, methods for diagnosis, and emergency protocols.

The following components make up the tool:

1. The probability that the risk event will occur in the future.

2. The qualitative assessment of the potential harm or health impairment that may ensue.

3. A comparative assessment of the risk based on a mix of probability and seriousness.

4. Determining potential corrective actions.

Procedure for Preliminary Hazard Analysis (PHA)

*Action to take:* The first set of requirements for PHA includes creating a PHA team, outlining the system that needs to be evaluated, and gathering risk information from earlier systems.

*Hazard identification:* Here, every potential hazard and unintentional event needs to be located. Right now, every component of the system needs to be taken into account. The recordings of all the findings are required.

Risks can be recognized in the following ways:

*Analyzing analogous current systems:* PHA involves the following steps: reviewing prior hazard analyses for systems similar to yours; reviewing hazard checklists and standards; thinking through energy flow within the system; thinking through inherently hazardous materials; thinking through interactions between system components; reviewing operation specifications; and taking into account all environmental factors.

*Estimating consequences and frequency:* We must assess the frequency and severity of each unintentional incident in order to establish the risk level. At this point, each hazard's frequency and consequence are taken into account.

*Risk assessment and subsequent actions:* This will make it possible to rate the occurrences and effects in a risk matrix. The steps that need to be taken in order to address the risk will depend on this rating level.

SWOT analysis

An approach known as SWOT (strengths, weaknesses, opportunities, and threats) analysis is used to evaluate a company's competitive position and develop strategic plans. SWOT analysis assesses potential future outcomes in addition to internal and external variables. A SWOT analysis is used to conduct a feasible, fact-based, data-driven study of the benefits and drawbacks of a firm, project, or industry as a whole. To ensure the correctness of the analysis, the organization must avoid gray areas and preconceived conceptions in favor of real-world events. It should be viewed as a recommendation to corporations rather than a hard mandate [11]. The pharmaceutical industry is a web of processes, endeavors, and establishments that find, create, and produce pharmaceuticals. Specialized production, preservation, and procurement make the pharmaceutical supply chain (PSC) more intricate. PSC encompasses all the operations that are involved in the discovery and development, production, distribution, and use of medications across a broad range of healthcare conveniences and other industries that support the smooth operation of these phases. An organization's strengths and weaknesses are evaluated in conjunction with external opportunities and threats through the use of the SWOT analysis method. By reducing the amount of information needed to improve decision-making, SWOT analysis reduces the complexity of strategic challenges and solves them effectively. SWOT analysis is also one of the most well-known and tried-and-true techniques for developing strategies.

Strengths

Minimal costs for labor, production, and clinical trials, extensive use of information technology (IT), robust interpersonal bond, strong backing from the government, the reverse logistics methodology.

Weaknesses

Long duration, risk associated with procurement, several manufacturing amenities, inadequate and intricate distribution network, insufficiently skilled labor force.

Opportunities

Growing population, greater likelihood of lifestyle-related disorders, opportunity for international trade, purchasing in partnership, growing disposable income.

Threats

Heightened rivalry, erratic demand, substandard supplier support.

But supply chain management (SCM) has proven difficult because of the ever-changing demands for short product lifetimes, the convergence of industry, and the dynamic circumstances that exist locally. A SWOT analysis is presented in Table 1.

**Table 1:** SWOT (strength, weakness, opportunity, threat) analysis

SWOT	Analysis
Strength	Skilled staff members
Weakness	Low profit margins
Opportunity	Global market
Threat	Competitors

Hazard operability analysis (HAZOP)

Because of the known impact of pharmaceutical products on human health, the industry is subject to some of the most stringent regulations nationally and internationally [12]. Standards of protection and effectiveness are critical for getting the best possible outcome under the correct treatment plan, as laws are used to ensure prevention, detection, treatment, or cure. As a result, pharmaceuticals have played an important role in the conventional industry in meeting the new criteria. The regulatory agencies support the implementation of new technology and manufacturing techniques play a significant role in this process. The research and management that is designed to lessen the potential harm that a nonconforming product could cause to health. This allows for the detection of undiagnosed difficulties during the medication research and production stages, as well as adverse responses, inappropriate uses, and therapeutic failures. There exist various approaches that have the potential to ensure product quality. Nevertheless, with respect to the manufacturing process, it is important to build a possible instrument that may result in a workable process risk analysis.

The HAZOP Procedure

Following the discovery of unacceptable risk or consequences, the HAZOP approach may need to be applied. In this case, a list of suggestions and steps to enhance the procedure or prevent hazards may be necessary:

*System description:* Defining the system's boundaries and scope is the initial stage.

*Arranging*: Determine the goals of the HAZOP analysis; create the schedule, worksheets, and other materials. For analysis, split the system up into smaller components. Analytical items need to be defined.

*Actions of an interdisciplinary team: *Choose a team leader and assign duties to each member. Every member needs to be an expert in a process-related technical field.

*Gather data:* Piping and instrumentation diagram (P&ID), process flow diagram (PFD), manuals, technical descriptions, and other technical data pertaining to the unit or process must be gathered and used for analysis.

*HAZOP delivery: *Identify the things that need to be assessed, specify the parameters, define the term "deviation" as a guide, and develop the causes, effects, and suggestions.

*Duties:* Include assigning accountability for carrying out measures to lower risk levels.

*Inspection:* Ensure that activities are carried out by reviewing the HAZOP suggested measures.

*File:* Keep track of the HAZOP procedure so that it can be analyzed further. Lastly, the HAZOP report that details the analysis carried out by the multidisciplinary team should be completed, incorporating all of the methodology's components.

Applications in the Pharmaceutical Industry

The HAZOP technique for industrial risk assessment necessitates a thorough comprehension of each component's function and relationship to the system as a whole. In order to fulfil quality standards, the industrial infrastructure in a number of areas needs to be modernized nowadays. The pharmaceutical and biopharmaceutical industries are one exception that stand out for their remarkable recent evolution.

**Figure 3:**
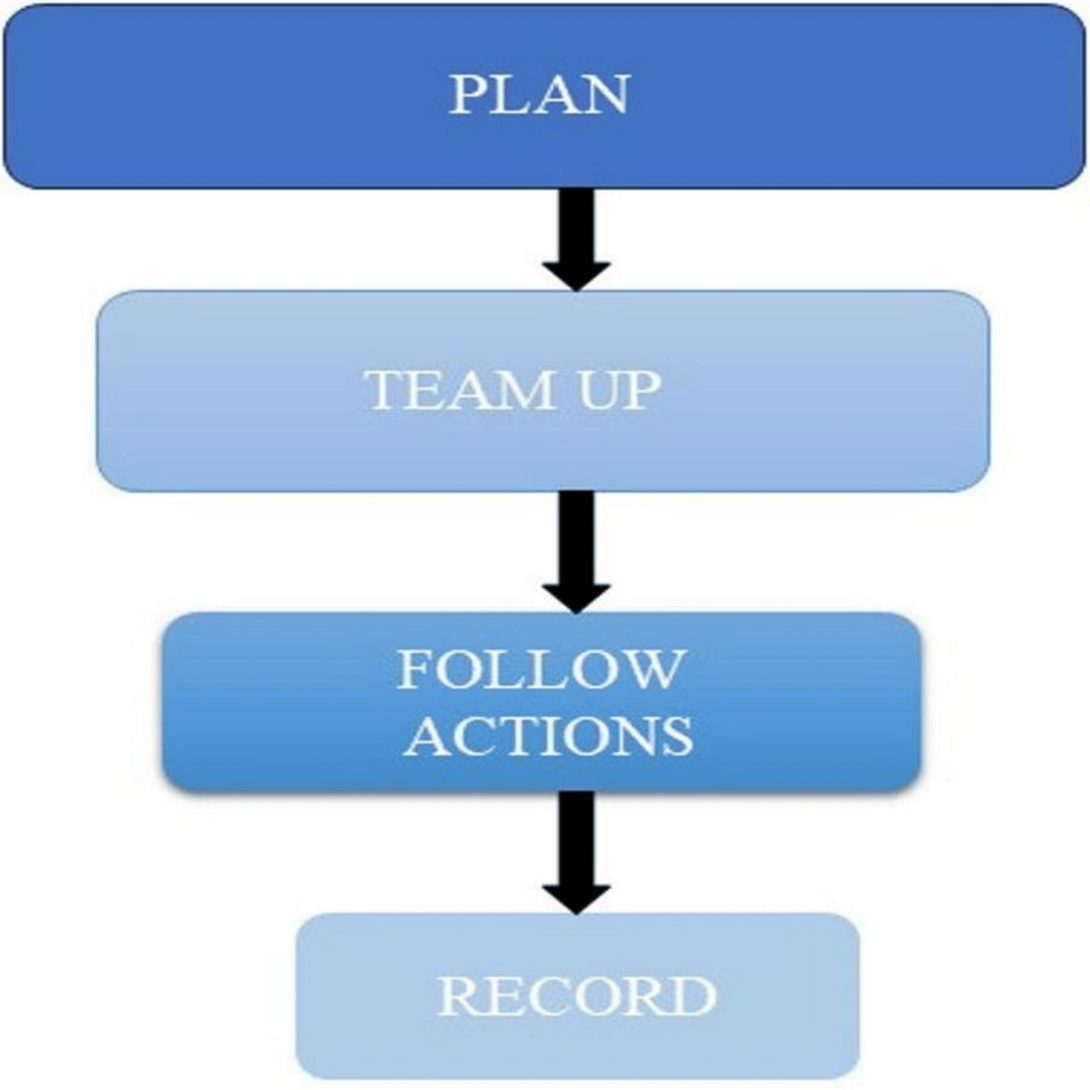
The flow pattern of HAZOP This image was created by Dr. Abdul M. Kaleem. HAZOP: Hazard operability analysis.

The flow pattern of HAZOP is given in Figure 3.

Root cause analysis (RCA)

Root cause analysis is a productive technique that lets you explore the fundamental causes of recurring problems in great detail. It assists you in going beyond merely managing symptoms and deals with the underlying issues that give rise to persistent issues. Through the identification and comprehension of these fundamental causes, you can execute efficacious remedies that avert subsequent reoccurrences [2,7]. The goal of root cause analysis (RCA) is to pinpoint the sources of issues in processes so that corrective measures can be taken. This component is usually absent from control charts; however, non-random patterns on the chart might serve as a basis. However, the process of identifying the cause or causes is challenging due to the vast number of potential relationships between patterns and causes. To make this procedure simpler, certain process data (at the moment of change) can be combined with chart patterns. For instance, if process data indicates that the operating machine has not been serviced in a while but that the material has recently been tested and found to be in good condition, there is a good chance that the operating machine's poor condition is what caused the issue. This is because pattern analysis can reveal whether the machine's condition or the quality of the input material is what caused the out-of-control situation. The linkage structure between assignable causes, process data, and chart patterns is illustrated.

The Importance of Finding Root Causes

It is crucial to identify and deal with a problem's underlying causes for a number of reasons. First of all, by addressing the root causes of the issue, it aids in preventing its recurrence. Moreover, RCA helps to reduce expenses. Pharmacies can increase their profitability and resource efficiency by reducing waste, rework, and downtime related to reoccurring issues. Addressing the underlying reasons also improves client happiness and the quality of the final product. We can make sure that our products always live up to client expectations and satisfy the highest standards by getting rid of the things that cause deviations or flaws.

Furthermore, RCA is a major factor in the continual improvement of industrial processes. We may make well-informed decisions to enhance and optimize our processes, leading to higher production and efficiency, by locating and resolving the underlying issues.

Statistical process control (SPC)

In the current competitive global market environment, one must be better than others and more competitive in order to thrive [13]. A business cannot expect to stay successful by basing its future success on the accomplishments of the past. Numerous well-known instances exist of businesses, both big and small, that were once industry leaders but are now essentially shells of their former selves. The use of statistical techniques for process monitoring and control to ensure a process runs as efficiently as possible to generate a conforming product is known as statistical process control (SPC). A process operates predictably under SPC in order to generate the greatest amount of conforming products with the least amount of waste. Design experiments, continuous improvement, and control charts are important SPC tools. It is feasible to find and fix process variances that may have an effect on the end product's quality, hence reducing waste and the risk of consumer concerns. SPC outperforms other quality approaches such as inspection because it focuses on problem avoidance and early detection.

SPC Implementations

Understanding and identifying crucial product attributes that are relevant to customers or crucial process variations is critical for SPC applications.

Key actions for applying SPC into practice are:

1. Determine the established procedures.

2. Determine what aspects of the process are measurable.

3. Describe the ways in which qualities naturally vary. 

When identifying differences resulting from unique sources, multivariate statistical process control (MSPC) can be more effective than traditional statistical process control. During World War II, control charts were used far more frequently in the United States to guarantee the quality of bombs and other strategically vital products. After the war, the usage of SPC techniques decreased slightly, but it was later highly utilized in Japan and is still in use today.

**Figure 4:**
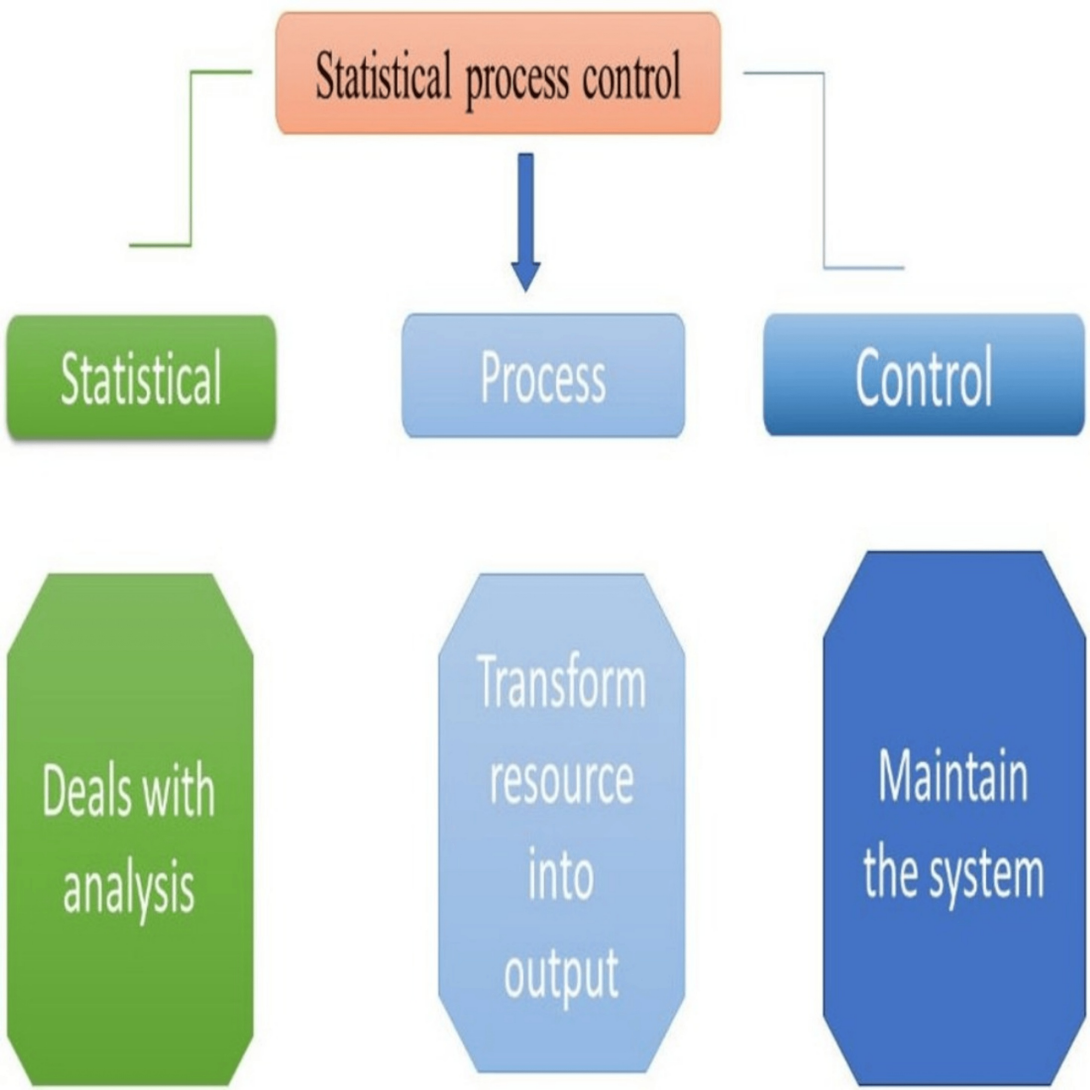
The principles of statistical process control This image was created by Dr. Abdul M. Kaleem.

In recent years, organizations all over the world have embraced a number of SPC methodologies, particularly as a part of quality improvement programs like Six Sigma. Statistical software packages and advanced data-gathering methods have played a major role in the broad use of control charting procedures. Control charts seek to discriminate between two forms of process variation: (i) variation occurs owing to common reasons that are basic to the method and will always be present and (ii) variation with a definite cause, which comes from outside sources, indicates that the method is not statistically controlled. A variety of tests may be employed to determine whether an unforeseen occurrence has occurred. However, the chance of an error increases with the total amount of tests performed. The principles of SPC are mentioned in Figure 4.

Process hazards analysis (PHA)

Toxic or combustible compounds are frequently used in batch reactions in the pharmaceutical industry. This form of processing may result in runaway reactions, fires, or explosions that endanger people and property. Any technique (HAZOP, What-If, Checklist, etc.) used to identify hazards and manage risk can only yield relevant results if it is supported by sufficient process safety information (PSI) and documentation. The Occupational Safety and Health Administration's process safety management (PSM) system does not apply to all pharmaceutical processes; however, the regulation serves as a good guide for PSM operations. Chemical reaction risks, including combustibility and toxicity of all the reacting agents, intermediates, and products, must be incorporated into PSI in the pharmaceutical industry [7,14].

Minimal Criteria for a PHA Program

The following are the minimal criteria for a PHA program:

1. Establishing a priority list and carrying out evaluations in accordance with the necessary timeframe.

2. Mapping and assessing the process hazards using a suitable approach.

Handling process hazards, past events with catastrophic potential, engineering and administrative controls relevant to the hazards, the fallout from control failure, facility location, human factors, and a qualitative assessment of potential effects on employees' safety and health in the event that a control failure occurs.

Performing a PHA involves using structured methodologies to assess the safety of a process, leveraging the engineering and operational skills to identify and mitigate the risks.

Putting in place a system to deal with findings and recommendations quickly guarantee that the recommendations are addressed and recorded, record actions taken, a written schedule for finishing actions created, and operating, maintenance, and other staff members who are involved in the process or could be impacted by actions are notified.

**Figure 5:**
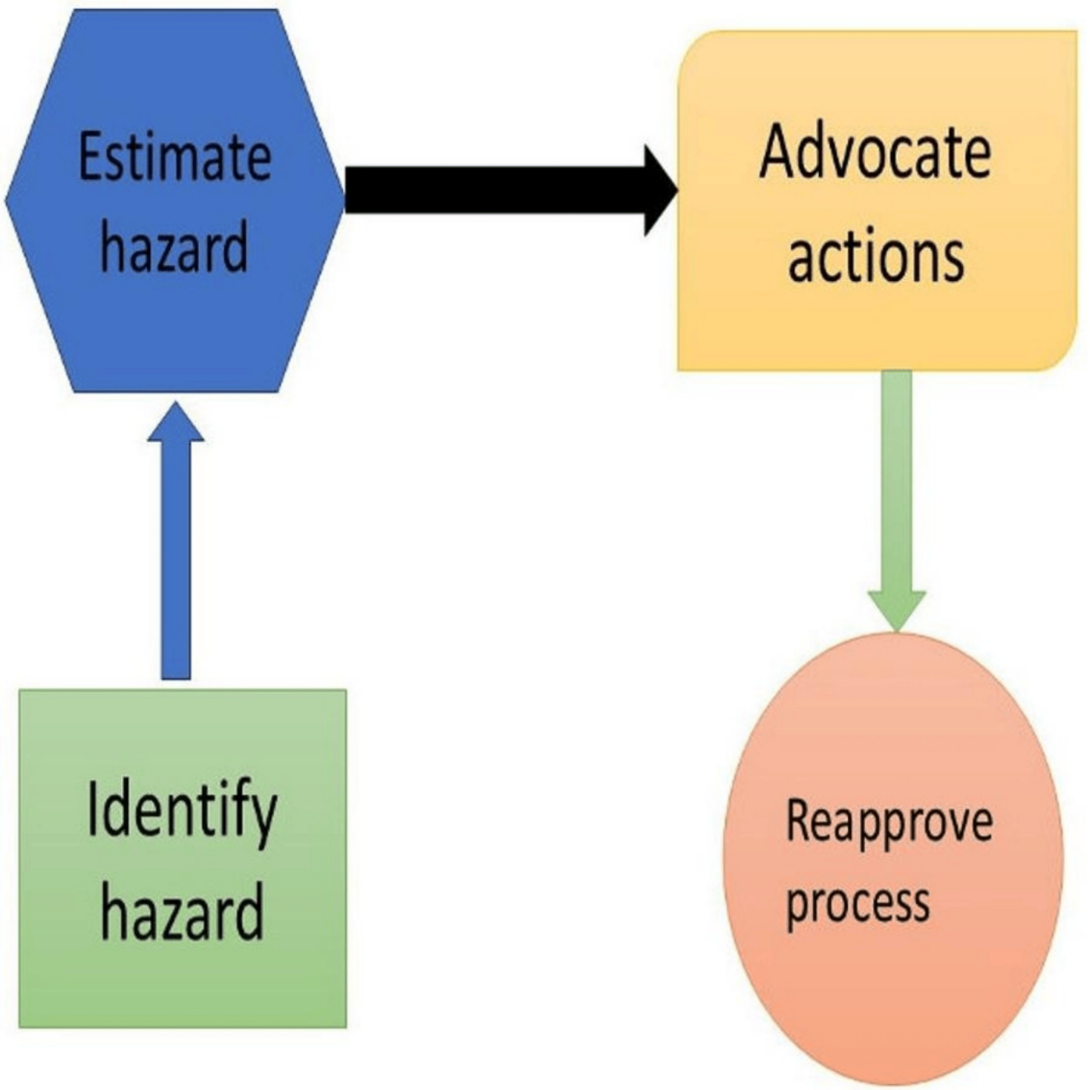
The steps of process hazards analysis This image was created by Dr. Abdul M. Kaleem.

PHAs should be updated and revalidated at least every five years. The PHA documentation and updates should be preserved over the duration of the procedure. The steps of process hazard analysis are mentioned in Figure 5.

The definition and their criteria of severity are listed in Table 2.

**Table 2:** Definition and their criteria of severity

Definition and the corresponding criterion for severity		
Severity	Scoring	Criterion
Critical	3	Manufacturing will be stopped
Major	2	Degree of hazard effects will cause products to differ
Minor	1	No effect on the product

Environmental risk assessment models (ERA)

Some pharmaceutical traces find their way into surface waterways and soils after the patients use them. The issues that were found were the basis for establishing the environmental risk assessment (ERA), implementing risk mitigation, boosting data transparency, bridging data gaps, and managing pharmaceutical manufacture. While some of the aforementioned flaws can be partially fixed by non-legislative measures (national initiatives or Europen Union (EU)-level projects), others call for modifications to the law as it is. As a result, one of the main goals of the Pharmaceutical Strategy for Europe is environmental challenges [15]. All active ingredients - aside from those that are found naturally in foods like vitamins, proteins, minerals, and amino acids - require an ERA. The evaluation process is a sequential one.

*Goal 1*: At the moment of marketing authorization (MA), the ERA is delivered on schedule and in full. To determine the appropriate mandated risk mitigation measures, a thorough ERA at the time of MA is required. If not, items risky to the environment could be released onto the market without the necessary risk mitigation procedures in place. 

*Goal 2*: In the event of danger, risk mitigation measures, such as frequent re-evaluation, are required. Only the applicant's assessment of the environmental risks and the justification for the safety precautions that must be taken for the storage, patient administration, and disposal of waste products are required per Directive 2001/83/EC art. 8.3(ca): "An assessment of the pharmaceutical product's possible environmental dangers. This effect will be evaluated, and particular plans to mitigate it will be considered on an individual basis." In actuality, this basically means making a declaration about how to dispose of the product, independent of the ERA's decision. These claims are included in the product characteristics summary, packaging, and labeling that are posted on national or European Medicines Agency (EMA) websites.

Supply chain risk assessment model 

Any dangers in the supply chain for pharmaceuticals have a chance to harm the patients' lives by stopping them from receiving prescriptions, as well as wasting money. Risk management is important not just in the supply chain of pharmaceuticals, but also in other pharmaceutical areas such as prescription and therapeutic use. In health systems, identifying and implementing risk management strategies for the supply chain of pharmaceuticals is critical. Because medication is a highly controlled product with tight requirements imposed by governmental regulatory authorities, managing risks is becoming more important [14]. Customers, suppliers, intermediaries, and outside service providers are all included. In addition, it covers all operations related to production, shipping, marketing, sales, product design, finance, and information technology. One of the most crucial elements of the modern supply chain is supply chain risk management. According to supply chain management principles, there are two types of risks in the supply chain: disruptive risks and operational risks. Even as the operational risk is less substantial than the disruptive risk, if it is ignored, it could have a big influence on the success of the business. This is the reason why it's critical that businesses and supply chains address operational risks. Hazards related to people, processes, machinery, and outside occurrences are all considered operational hazards. Since these risks are never completely eliminated, businesses must take steps to reduce them. Risk mitigation in the global pharmaceutical supply chain has grown in importance at the business executive levels. Particularly the pharmaceutical industry is distinguished by its highly technical core functions, which include development and manufacturing. Other unique characteristics of this industry set it apart from other sectors, including product perishability, the significance of safety and security, and regulations. Pharmaceutical companies confront several risks as key players in the medicine supply chain. These risks impair the supply of medicines in a variety of ways, involving quantity, quality, and on-time distribution to the proper clients and places. As a result, it is strongly recommended that risks be recognized and dealt with throughout the pharmaceutical company's supply line [11]. In pharmaceutical supply chains, outsourcing logistics entails a number of risks. Organizations need to take proactive risk management measures in order to stay competitive. Risks must be identified and evaluated in order to ensure it happens.

Process analytical methodology (PAT)

In order to achieve strict standards for product quality and remain competitive, the pharmaceutical sector is seeing a growing demand for the introduction of new and efficient production techniques that can produce consistently high-quality goods. Historically, pharmaceutical goods have been produced using batch procedures with predetermined operating parameters and set points. In these kinds of procedures, proving process repeatability and testing to satisfy predetermined acceptance criteria usually form the basis of the drug substance control strategy. This method, however, is frequently associated with product faults, rejections, and recalls and is known to cause batch-to-batch variations in product quality. A major factor contributing to process development issues is the intense competition to launch new items quickly onto the market [16].

Pharmaceutical QbD is a systematic approach to development that begins with predefined objectives and emphasizes process understanding, methodical preparation, design space specification using prior knowledge, or experimentation supplemented by sound science principles. Several reviews focusing on the principles of QbD in pharmaceutical manufacturing have recently been published as well. Process analytical chemistry equipment, multidimensional data gathering and analysis, process control and monitoring, continuous procedure efficiency, and information management are all critical PAT components. There has been a lot of interest in the application of PAT among academics and industry since the Food and Drug Administration (FDA) released a draft advisory in 2004 asking the industry to accept PAT in pharmaceutical manufacturing. The main goal of process analytical technology is to decrease process variability. Product variability should be decreased by integrating automated feedback control of the process parameters affecting essential quality attributes with online measurement and/or modeling of these attributes. Lowering product variability will consequently lower the chance of commercializing off-spec products. Reduced cycle times and improved process efficiency also have advantages (e.g. enhanced yield, throughput, and equipment utilization).

Application

Process analytics can be used at every stage of the manufacturing value chain, from the creation of active pharmaceutical ingredients (APIs) to the production of pharmaceutical products. Ancillary operations like waste stream monitoring and buffer and reagent preparation for use in the manufacturing process can also benefit from the application of process analytics. The latter could offer crucial details about which process steps could be enhanced in order to reduce losses, raise production yields, and enhance process greenness. Pharmaceutical detection levels in rivers are becoming a growing source of concern, leading to widespread use of process analytics.

Layer of protection analysis (LOPA)

The International Electrotechnical Commission's (IEC) 61511 standard, for example, has emerged as an international standard for control systems for computer-controlled processes. The difficulty of meeting these criteria regularly led to the establishment of the layer of protection analysis (LOPA), which establishes the requisite safety integrity levels (SILs) for computerized security processes in chemical industry production facilities. The Centre for Chemical Process Safety (CCPS) in the United States was the initiator and promoter of these requirements. A more straightforward method of quantitative risk assessment called LOPA has been suggested. This is made possible by multiple modules in the Accident Risk Assessment Methodology for Industrial Safety (ARAMIS) technique. One possible way to complete the ARAMIS barrier assessment is using LOPA. This strategy is frequently employed and can change based on the user company's attitude and corporate norms. As a result, there will be changes from one establishment to another that stakeholders might find unacceptable. It's possible that some businesses are unable to establish corporate standards. It is obvious that the selection process is simpler in situations when a national regulator establishes guidelines for acceptable individual and/or society risks [2]. True independence can be validly doubted thanks to the study method and its guidelines. This may result in certain simple and affordable system upgrades where hardware or software is already installed but whose "architecture" does not guarantee independence. In general, barriers that halt the scenario and bring the process back to a safe state are simple for LOPA users to evaluate. These obstacles might be disregarded by the cautious operator. On the other hand, one could counter that they make the situation less serious. In addition to addressing the independence issue, the ARAMIS approach provides a framework for evaluating the effectiveness of each barrier by analyzing its response time, effectiveness, and probability of failure on demand. This holds true for the organizational barriers, software, and hardware taken into consideration. The methodical treatment of these issues in, say, the management system audit should aid the LOPA user in improving his evaluation of them and enable them to be suitably taken into account in his analysis. Additionally, the ARAMIS method explicitly tackles the sensitivity and uncertainty issues that certain authorities demand and that are an essential part of objective analysis. Although some LOPA users may already have established methods for incorporating them in their studies, adopting the tools in ARAMIS may make their process easier and result in greater consistency. The ARAMIS method's scope is broader than that of LOPA, and it provides a means for LOPA users to "close the loop" by evaluating sensitivity and risk mapping. Both LOPA and ARAMIS can identify weaknesses in the systems and offer solutions for a practical and affordable approach to fix them. When identifying and characterizing hazards as part of a HAZOP, LOPA frequently comes after a qualitative risk analysis. Owing to its simplicity of use, LOPA has gained popularity as a screening method or substitute for QRAs. Moreover, a streamlined risk assessment methodology that uses LOPA to evaluate hazard barriers is used in the unintentional risk assessment methodology for industries (ARAMIS) methodologies supported by the European Commission.

Risk matrix

Risk matrices are useful tools for prioritizing and rating the risk of (typically unwanted) events, as well as determining if a given risk is acceptable. A risk matrix is a graph that depicts the key elements of a negative event, including "consequence" and "the probability," as well as the general concept of risk. It makes use of separate risk, chance, and consequence categories. To stress the potential for error in risk announcements, both risk experts and non-specialists prefer to use classifications rather than numerical quantities. A handful of risk categories, usually represented by different colors, are assigned to the mixtures of outcome and likelihood. This conversion may take into account negligible characteristics, such as substantial hazard aversion [7]. It is clear from examining risk matrices’ implementation that this simplistic instrument has some significant drawbacks. These flaws should be recognized by both risk matrix designers and users, who should also make sure that the risk matrices are used correctly and lead to the right conclusions. Some publishers have only recently addressed this issue. There are primarily two uses for risk matrices. When debating the appropriateness of risk matrices, the application or goal of the risk matrix is pertinent. Making decisions about whether to accept risk is one use; prioritizing which risks should be taken care of first is another. Often, when it comes to risk acceptance, there are only three distinct categories of risk: risks or occurrences that pose an unacceptable risk (typically denoted by the color red); risks or occurrences where the risk is deemed to be "broadly acceptable," meaning that no more risk reduction is necessary (usually shown in green), and an intermediate level where risk reduction should be done "As Low as Reasonably Practically" (ALARP, frequently represented in yellow). It is not necessary to further prioritize hazards in light of these interpretations, at least not in the red and green sectors.

Applications

It is necessary to employ discrete categories or categories for probability and result when applying a risk matrix. Some authors have questioned this feature, but it should be emphasized that subjective evaluations must be made for any risk assessment that isn't based solely on statistical data and a mathematical consequence assessment. As such, the problem is more complex than just how the risk matrix should be applied. We recognize that a priori estimates of the probability and impact of unfavorable events are not exact; rather, they are subjective estimates that are rarely corroborated by statistics or observations. There are several strategies to steer clear of subjective prejudice. The first is to minimize the need for subjective judgments by using quantitative data as much as feasible. By using quantitative descriptions (ranges, reference points) in the defining of categories, variability in comprehension of spoken descriptions can be reduced. Extending the range of the categories (i.e., adding an additional category) to both sides of the predicted range of consequence and likelihood can mitigate centering bias.

Leopold matrix

Leopold created a matrix in 1971 to assess the effects on the environment. This matrix is made up of rows that reflect environmental factors like air and water quality and columns that represent organizational actions like drilling, blasting, trucking, and mineral processing. Leopold matrix can also be used as a supplment for ERA. The matrix is built with columns signifying different organizational operations and rows signifying environmental aspects like emissions to the air and discharges to water bodies. Each activity's related cells are examined in light of the potential aspects. The reference value is the highest score among them. Every factor that scores higher than 50% of the reference value becomes relevant and is given a corresponding ranking. These are used to evaluate the total environmental risk [17]. Environmental impact assessment practitioners can systematically rank potentially significant environmental cause-and-effect interactions using the matrix, which offers an organized framework. Furthermore, the grid format makes it possible for results to be visually shown in a way that the general public and policymakers can easily understand. The matrix can also be made to grow or contract according to the size and environmental setting of a particular project, making it useful for both large- and small-scale initiatives. Lastly, the tool's ability to be used at different points of time during the environmental impact assessment process is helpful to practitioners.

Safety integrity level (SIL) assessment

The safety requirements of the SIL are categorized into four distinct levels, with SIL 1 representing the least stringent and SIL 4 the highest. For the safety functions carried out by safety systems, these levels are utilized to define the safety integrity requirements. For every SIF, the target SIL shows the relative importance of the safety standards [18]. SIL determination can be done in three ways: semi-quantitative, quantitative, and qualitative. There are various approaches that fall under these categories, each with pros and cons. A safety instrumented system (SIS) that uses a SIF is said to provide functional safety. It is necessary to first establish and then confirm SIL targets. In order to safeguard people, the environment, and property from dangerous occurrences like explosions caused by excessive pressure or product spills from elevated tank levels, SIS are extensively utilized in the process sector. To deliver a given degree of risk reduction, the SIS needs to meet a number of safety conditions. Numerous recommendations and standards that specify the safety instrumented function (SIF) requirements as well as the way the SIL should be determined and its needs met have been produced. Choosing the necessary SIL for a SIF is referred to as SIL determination. Following the risk assessment, the requisite SIF must be determined, put into practice, and confirmed to meet the SIL requirements in order to reach the desired level.

PESTLE analysis

Although the pharmaceutical industry has existed for hundreds of years, most medical conditions have only recently been understood and treated in the last century. The pharmaceutical sector has a mixed future outlook, despite being quite profitable for those who work in it. The political, economic, sociocultural, technological, legal, and environmental (PESTLE) elements that will determine the future of this business will be examined in this PESTLE analysis [19]. Due to sophisticated technologies, the use of raw materials, and insufficient recycling, the pharmaceutical industry faces environmental issues. The manufacture of active ingredients results in notable environmental consequences due to the increased usage of water and the generation of effluent. The primary environmental consequences include excessive consumption of natural and chlorinated solvents for organic substances, inorganic salt, ammonia-ammonium particles, metallic substances, and discharges of adsorbable organic halogens. These emissions of these chemicals have an adverse effect on biodiversity and the quality of the air and water.

Regulatory frameworks

Almost every country has strict regulations governing the pharmaceutical industry. The regulatory structure establishes key criteria for carrying out business in the pharmaceutical sector. It is comprised of several governmental agencies and various drug-related regulations. This mainly refers to the reliability and health of drugs. It may also have an effect regarding where and in what way prescription drugs are sold. In any case, existing drug manufacturers and suppliers have it considerably easier thanks to this complex regulatory framework, which creates an important obstacle to entry for new competitors in the pharmaceutical industry.

Health Trend

Considering older people and rising obesity rates, there is a trend toward increased health consciousness, particularly in developed countries. Healthy living is becoming increasingly popular among young and middle-aged people. Good habits are increasingly trendy right now, and they involve eating well, exercising regularly, and even cultivating mindfulness. In future years, fewer medicinal goods may be necessary if this trend continues and more individuals choose healthier lifestyles.

Political Factors

A politically secure setting is required for any organization to thrive. Some of the political scenarios could have an impact on the pharmaceutical industry. The majority of countries maintain frameworks that include regulations on certification, security necessities, and other issues. They also detect forbidden substances that are harmful to health. If a pharmaceutical company ignores these guidelines, its operations may suffer significantly. In order to make drugs more inexpensive for the general public, administrations in the majority of nations work to restrict drug prices. It can hinder the expansion of pharmaceutical businesses.

Economic Factors

Any type of economy has an immediate influence on businesses. When a country's economic conditions change, the pharmaceutical industry is also affected. The PESTEL study can help the pharmaceutical industry assess the financial circumstances that may affect its companies. The public's household incomes are increasing in step with the country's changing economic conditions. It could help them receive some critical meds. They may feel driven to buy more expensive prescriptions, which were formerly out of range for many individuals.

Social Factors

Any country's sociocultural characteristics can have an impact on its auxiliary industries. The pharmaceutical industry, like all others, is heavily influenced by sociocultural factors. The following sociocultural factors may influence the rate at which the pharmaceutical industry expands: People's lifestyles are both highly quick and stagnant. As a result, more people are growing obese. Consequently, dealing with disorders such as type 2 diabetes, the thyroid, and elevated blood pressure has become common. To address this, patients must take medication on a regular basis. As an outcome, pharmaceutical companies' sales are also increasing.

Technological Factors

Technological advancements have a significant influence on the pharmaceutical industry. The pharmaceutical sector's PESTEL research can show how technical issues can affect operations: The pharmaceutical industry depends largely on technology. Pharmaceuticals with inexpensive manufacturing and high quality have been developed as a result of research and biotechnology developments. More people will be able to get drugs that they had previously been unable to afford. The drugs require the proper storage conditions. Technology has made it easier to transport and store pharmaceuticals without risking deterioration in harsh circumstances. Owing to technology, pharmaceutical companies may now execute campaigns that reach a bigger number of companies. Furthermore, they can deliver the drugs straight to the door, broadening each business's market reach.

Environmental Factors

Environmentalists are deeply worried about the environmental impact of waste products. A number of environmental issues may have an influence on the pharmaceutical industry. Several countries are proposing rules to reduce the environmental impact of pharmaceutical production, which has a high carbon footprint. The production of pharmaceuticals produces a variety of biotechnology pollutants; new enterprises could struggle to establish themselves due to the cost of compliance with these regulations. They may constitute a health danger to people. To guarantee everyone's safety, the firm must remove this rubbish.

Legal Factors

The pharmaceutical industry is not considerably influenced by the nation's legal framework. However, unexpected repercussions may limit the pharmaceutical industry's progress. The PESTEL analysis of the drug industry may help identify the legal variables that could contribute to the market's growth.

Scenario analysis

Future research and development (R&D) productivity is expected to be higher, according to some industry experts, because of innovation and new technologies like digitalization [20]. However, data indicates that productivity has not always grown as a result of the new technology introduced in the 1970s and 1980s, such as computers and other information technology (IT) gadgets. Robert Solow, the recipient of the Nobel Prize in Economics, famously said, "You can see the computer age everywhere but in the productivity statistics." The FDA was the first regulatory body to acknowledge that, in addition to accommodating the constraints on funding clinical trial programs, modernizing drug development was necessary to secure future investments in R&D. Due to the high failure rates in clinical research, the productivity of pharmaceutical R&D investments is decreasing. Adaptive designs can reduce costs and increase efficiency in medication development, as the US FDA recently acknowledged. In addition to estimating the influence on worldwide R&D expenditures and potential outcomes expressed in life years gained, our goal is to model cost-saving impacts. Low productivity in R&D was said to put at risk the pharmaceutical industry's fundamental business paradigm. "The pharmaceutical industry's viability (let alone its chances for continued growth), at least in its current form, is in considerable peril without a substantial rise in R&D productivity.” Future R&D productivity is expected to be higher, according to some industry experts, because of innovation and new technologies like digitalization.

Risk register

Since the publication of ICH Q9 in 2005, the pharmaceutical industry has increasingly used a quality risk management (QRM) method to assess and handle quality concerns within their organizations. ICH Q9 provides a structured framework for detecting, assessing, and reducing pharmaceutical quality concerns. By incorporating QRM principles into their daily operations, manufacturers can proactively detect potential quality concerns, prioritize them considering their potential impact, and adopt effective risk management strategies. The part of the ICH Q9 QRM life cycle that focuses on sharing information about risk between decision-makers and others, such as departments and sites throughout a company, across industry and the patient, and between industry and regulators, may become more difficult as a result of this growth in risk data. Risk registers are an effective management tool that help make sure decision-makers have the essential data they need to do their jobs [17]. Parenteral Drug Association (PDA) Technical Report 54 states that companies shouldn't build their QRM solutions exclusively using risk registers. A database of hazards that reach or surpass a predetermined risk limit and affect a specific scope or organization is necessary. This index comprises risks that currently affect the organization or scope, including risks that have planned or active mitigation activities and risks that cannot be mitigated; all of these risks have been assessed, approved, and (at the very least, temporarily) authorized by senior management. The Medicines and Healthcare Products Regulatory Agency's (MHRA) frequently asked questions (FAQ) are also recommended as a management process be set up to review the risk management and this process should be integrated into the review of quality management. This suggestion is similar to one found in the PIC/S Aide-Memoire on Review of QMS Execution, which says that ICH Q10 provides a model for management review. It helps to have a risk register or something similar so that the inspector and the company may review it together. In a similar vein, opportunities and risks must be included in management review meetings even though ISO 9001 does not necessitate the usage of a risk register.

Dynamic risk assessment

Dynamic risk analysis (DRA) is a complete, continuous real-time process for updating and incorporating changing levels of risk into the whole risk profile in response to rapidly changing circumstances. The limitations of standard and traditional quantitative risk assessment approaches, which are essentially static and unable to update the entire risk profile or estimate risk dynamically, can be solved by developing and using new DRA techniques [2]. Since the beginning of the 1980s and 1990s, numerous dynamic methodologies for incident sequence evaluation in context with time-dependent human behavior and actions, as well as system reliability analysis, were created. Over the past few decades, research on dynamic risk analysis and management methodologies has advanced significantly in order to consider dynamic risk measurements in contexts or circumstances that are continually changing. It offers a thorough and current overview of several risk assessment techniques, their development into dynamic approaches and applications, as well as current developments, opportunities, and constraints. Additionally, the writers offer several recommendations for additional study. The first and most important step in the risk analysis process is hazard identification. Not all threats can be identified using conventional and basic approaches such as checklists and what-if analyses. This is because some methods have limitations, others require experience, and still others lack the necessary knowledge or information.

Major Drawbacks

Operators and system elements communicate with one another and react to disturbances in a dynamic way. They talk about the main shortcomings of traditional models used to handle the emergence of complicated scenarios that are heavily impacted by the incorporated dynamic reaction brought about by the operator's improper task performance or actions during an accident. The majority of the dynamic event analysis techniques covered above are more complicated and need a large amount of data input. In order to determine which of these approaches was best for use in risk analysis in real time, they also evaluated the others. When working with complex systems, all analytical techniques first demand an explicit characterization of nearly every state and transition of the system, which leads to very large models. The main flaw in the analytical approaches is that the operator's mental state is not explicitly represented by a system state specification. However, system states can be precisely defined by simulation approaches as the result of a particular set of scenario-based modeling principles.

Decision tree approach

Decision trees are a basic but effective tool for developing models for predicting structure-activity relationships. A classification model is employed when the variable that responds is categorized, such as active/inactive, and a regression model when the response's variable is continuous. The dataset is recursively divided into two or more subsets by the approach according to the value of a molecular property like log P or the presence or absence of a structural feature. The three main components of the analysis are the following rules: (i) dividing each node in the tree into a subset; (ii) defining when a node may no longer be split into subsets; and (iii) simplifying or pruning the original tree [8]. Several advantageous properties of decision trees make them suitable for constructing prediction models in pharmacological research:

DTs can manage exceedingly large, structurally diverse compound collections;

DTs can handle large feature sets, which may comprise a mixture of constant, discrete, and categorical data;

DTs have the ability to disregard irrelevant descriptors;

They have shown helpful in elucidating a complex, nonlinear response;

DTs offer an easily comprehensible decision route for comprehending the prediction of a test substance, and it is simple to analyze wrong predictions. Contrasts the benefits and drawbacks of tree-based approaches with those of other statistical learning techniques. When compared to other learning approaches, the prediction of accuracy of decision trees is comparatively low, which is their main shortcoming. Several improvements have been proposed to enhance the prediction capacity of decision trees maintaining their favorable characteristics. A decision tree can be charged to any other compound that has the required descriptors once it has been built from a training set.

Systematic risk identification and assessment

Pharmaceutical enterprises that rely on research encounter additional hazards due to expanding research and development expenses; as a result, risk management has emerged as a previously unknown profession for industry executives [17]. Pharmaceutical firms encounter numerous obstacles, but they also benefit when they take cautious risks. A thorough, organized, and complex framework for risk identification and assessment is obviously required, even though specific concerns (such as medication safety or difficulties recruiting participants for clinical trials) have been identified and incorporated into contemporary pharmaceutical R&D activities. Avoiding or reducing risks' likelihood and/or occurrence is the goal of risk management in research and development (R&D) in order to better accomplish goals and, as a result, increase R&D efficiency [6].

*Risk 1:* The two main approaches to mechanism/indication pairing and indication selection are backward/reverse translation and forward translation.

*Risk 2:* Insufficient clinical trial planning includes dose level picking and the dosage schedule (risk of insufficiently or over-dosing), duration of study and number of participants (risk of underpowering or oversizing/time delay), research endpoint selection (risk for late-stage trials: the endpoints are not consistent with well-being authorities), and research cohort selection (inclusion/exclusion criteria that are excessively broad or too narrow for capturing disease activity and heterogeneity).

*Risk 3:* Inadequate patient acceptance and compliance, low enrollment rates, high screening rates of failure, early trial cessations, and inadequate risk monitoring approaches are all instances of poor clinical trial execution. 

The applications, advantages, and disadvantages of various risk assessment techniques are listed in Table 3.

**Table 3:** Applications, advantages, and disadvantages of various risk assessment techniques.

S.No	Risk Assessment Techniques	Advantages	Disadvantages	Application
1	Failure Mode and Effects Analysis (FMEA)	Proactive risk identification, root cause analysis, quality improvement, and systematic approach	Overemphasis on severity, difficulty in quantification, resistance to change	Supplier quality management, product development, process Improvement
2	Failure Mode, Effects, and Criticality Analysis (FMECA)	Improved decision-making, proactive risk management, cross-functional collaboration, and enhanced risk prioritization	Data availability, limited predictive power, and resource intensive	Process improvement, maintenance planning, and risk management
3	Hazard Analysis and Critical Control Points (HACCP)	Systematic methodology, enhanced consumer confidence, and risk reduction.	Risk of overreliance, limited scope, complexity	International trade, hazard identification, and measures.
4	Qualitative Risk Assessment	Standardization, objective evaluation, risk reduction, and safety integrity level	Maintenance, subjectivity, cost, complex process	Safety certification, safety instrumented system design, and process safety management
5	Quantitative Risk Assessment	Risk prioritization, precision, and comparability	Assumptions and uncertainties, data requirements, and limits	Drug development, supply chain management, and clinical trials
6	Quality Risk Management (QRM)	Efficiency, proactive approach, continuous improvement	Integration challenges, regulatory expectations	Change control, manufacturing operations, and product development
7	Event Tree Analysis (ETA)	Structured approach, probabilistic analysis, risk identification, and visual representation	Data requirements, complexity, assumption sensitivity, and resource intensive	Risk assessment and management, safety engineering
8	Bowtie Analysis	The linkage between the barriers and the system for safety management can be made clear, and various potential consequence outcomes are described.	Fails to offer a quantitative evaluation of risk, misleading sense of security, conflating threats with escalation factors	Emergency preparedness and response, incident investigation
9	Preliminary Hazard Analysis (PHA)	Cost savings, integration with design process, facilitation of risk communication	Limited scope, complexity of analysis, and potential for overlooked hazards	Process development, product development, regulatory compliance, and system design
10	SWOT Analysis (Strength, Weakness, Opportunity, Threat)	Comprehensive evaluation, strategic planning, and simple and easy to use	Limited focus, lack of prioritization, static analysis	Organizational development, project management, and market research
11	Hazard Operability Analysis (HAZOP)	Comprehensive hazard identification, operational improvements, early detection of hazards and multi-disciplinary collaboration	Risk communication challenges, documentation burden, time constraints, and limited scope	Process modification, safety management, risk assessment, and process design
12	Root Cause Analysis (RCA)	Improved decision-making, enhanced organizational learning	Implementation challenges, difficulty in identifying all factors	Structured approach, detect non-conformity and implementation of effective corrective
13	Statistical Process Control (SPC)	Quality improvement, data-driven decision making, early detection of problems	Overreliance on data, resistance to change, and complexity	In service industries, supply chain management, and construction and engineering
14	Process Hazard Analysis (PHA)	Risk reduction, process optimization, decision support, and identification of hazards	Dynamic nature, limited predictive power, resistance to change	Process safety management, new process design, emergency response planning
15	Environmental Risk Assessment Models (ERA)	Predictive capability, standardization, cost efficiency, data integration, scenario analysis	Validation challenges, uncertainty, limited scope	Risk communication, research and development, regulatory compliance
16	Supply Chain Risk Assessment Models (SCRA)	Quantitative analysis, risk identification, collaborative risk management	Assumption sensitivity, model validation, resource intensive.	Risk mitigation planning, strategic decision-making, supplier selection, and management
17	Process Analytical Technology (PAT)	Real-time surveillance, reduced variability, and waste facilitates QbD	Integration with existing systems, validation, and qualification	Real-time release testing, supply chain optimization
18	Layer of Protection Analysis (LOPA)	Simplified risk assessment, consistency, and transparency	Limited quantification, assumptions of independence	Hazardous operations planning, risk communication
19	Risk matrix	Visual representation, simple and intuitive, prioritization of risks and facilitates decision making.	Limited detail, lack of consistency, difficulty in quantification and subjectivity	Enterprise risk management, financial risk assessment, compliance, and regulatory risk management
20	Leopold Matrix	Structured approach, visual representative, decision support	Inflexibility, data requirements, subjectivity, limited	Project planning, stakeholder engagement, decision support
21	Safety Integrity Level Assessment (SIL)	Risk reduction, standardization, objective evaluation, and continuous improvement	Cost, maintenance requirement, subjectivity	Process safety management, safety instrumented system design, and risk management
22	PESTLE Analysis (Political, Economic, Social, Technological, Legal, Environmental factors)	Comprehensive analysis, holistic perspective, opportunity identification, and strategic planning	Overemphasis on external factors, limited predictive power, and data availability	Market research, business expansion, strategic planning, and regulatory compliance
23	Scenario Analysis	Strategy planning, innovation and creativity, risk management	Data limitations, resource intensive, overlooking black swan events	Risk management, policy development, and advocacy
24	Risk Register	Structured governance, communication facilitation, linkage to risk assessment	Complex procession, risk register ownership, complex processes	Quality system integration, communication tool, management review
25	Dynamic Risk Assessment	Risk awareness, quick thinking, encouraging proactive safety and comfort	Time-consuming, potential for inconsistency, potential for oversight	Enhancing safety protocols, improved risk management
26	Decision Tree Approach	Interpretability, identify important features, handling missing values and versatility	Instability, bias towards features with many levels, and difficulty with XOR (exclusive or) problems	Regression, anomaly detection, feature selection, and decision analysis
27	Systemic Risk Identification and Assessment	Comprehensive risk analysis, enhanced quality management, efficient resource allocation	Complexity, cultural shift, continuous monitoring	Supply chain integrity, clinical trial management, drug development

Future perspectives

The pharmaceutical industry's risk assessment is about to undergo a major shift as a result of the incorporation of cutting-edge methods and QbD concepts. This integration promises to enhance the excellence of pharmaceutical research and manufacturing by encouraging a more robust, proactive approach to quality and risk management. Process analytical technology (PAT), big data analytics, and real-time analytics are examples of advanced analytical technologies that can be used to improve process monitoring and identify changes that may affect product quality. By incorporating these technologies into the QbD framework, greater insights into process dynamics will be possible, leading to more accurate control and early risk detection. Artificial Intelligence (AI) and machine learning (ML) can be used to forecast possible dangers and real-time process optimization. Large data sets can be analyzed by these technologies to find correlations and patterns that conventional analysis might miss. AI-driven models can facilitate adaptive control strategies and dynamic risk assessment, guaranteeing real-time quality assurance and ongoing development. It will be essential for pharmaceutical companies, government agencies, educational institutions, and technology suppliers to work together. Advanced risk assessment and QbD methods will be developed and implemented more quickly through the sharing of best practices and expertise. Platforms for collaboration and consortia have the power to spur innovation, standardize methods, and guarantee that breakthroughs are widely embraced in the sector. The combination of cutting-edge methods and QbD principles will determine the direction of risk assessment in the pharmaceutical sector going forward. This synergy will provide a better understanding of processes, more efficient risk management, and improved product quality, which will propel pharmaceutical research and manufacturing excellence. The pharmaceutical industry's pursuit of optimal outcomes will increasingly depend on the unification of risk assessment and QbD as technology breakthroughs and regulatory frameworks undergo constant evolution. In addition to ensuring public health, this proactive strategy encourages innovation and operational effectiveness in the quest for pharmaceutical excellence.

## Conclusions

In conclusion, this review helps in understanding the importance of achieving excellence in quality, safety, and regulatory compliance within the pharmaceutical business requires unifying risk assessment approaches. Throughout the drug development lifecycle, a thorough awareness of potential risks is fostered by the integration of multiple approaches, such as failure modes and effects analysis (FMEA), hazard analysis and critical control points (HACCP), and fault tree analysis (FTA). Pharmaceutical businesses can improve their capacity to detect, evaluate, and reduce risks by utilizing the advantages of each strategy. This synthesis ensures compliance with strict international standards, enhances patient safety and product quality, and expedites regulatory filings and audits. Adopting a uniform methodology for risk assessment also encourages a proactive culture as opposed to a reactive one. These procedures will be further optimized by future developments in artificial intelligence, machine learning, and analytical tools, enabling real-time monitoring and predictive analytics that proactively address possible dangers. A comprehensive, dynamic risk management framework that is more sensitive to the intricacies of pharmaceutical manufacturing will be produced by this technological synergy. Regulatory organizations' attempts to promote innovation and standardize norms will help these integrated techniques become widely adopted. Industry-wide cooperation will be essential for promoting knowledge exchange and standardizing best practices. To sum up, the integration of risk assessment methods with quality by design (QbD) concepts will result in notable enhancements to pharmaceutical quality and safety, fostering ongoing improvement and optimizing operational effectiveness. In addition to guaranteeing adherence to strict regulatory requirements, this integrated strategy places the pharmaceutical industry in a better position to address the changing demands of public health, which will ultimately result in safer, more effective products.
